# Implementation Rate of Physical Rehabilitation in Neonatal Intensive Care Units in Japan: A Retrospective Observational Study

**DOI:** 10.3390/medicina60071075

**Published:** 2024-06-30

**Authors:** Yuto Ogata, Ryutaro Matsugaki, Manami Zaizen, Satoshi Kuhara, Keiji Muramatsu, Shinya Matsuda, Shutaro Suga, Hideaki Ito, Satoru Saeki

**Affiliations:** 1 Department of Rehabilitation, University Hospital of Occupational and Environmental Health, Yahatanishi-ku, Kitakyushu 807-8556, Japan; 2 Department of Work Systems and Health, Institute of Industry Ecological Sciences, University of Occupational and Environmental Health, Yahatanishi-ku, Kitakyushu 807-8556, Japan; 3 Department of Preventive Medicine and Community Health, School of Medicine, University of Occupational and Environmental Health, Yahatanishi-ku, Kitakyushu 807-8556, Japan; km@med.uoeh-u.ac.jp (K.M.);; 4 Department of Pediatrics, University of Occupational and Environmental Health, Yahatanishi-ku, Kitakyushu 807-8556, Japan; 5 Department of Rehabilitation Medicine, University of Occupational and Environmental Health, Yahatanishi-ku, Kitakyushu 807-8556, Japansae@med.uoeh-u.ac.jp (S.S.)

**Keywords:** very-low-birth-weight infants, neonatal intensive care unit, rehabilitation, diagnostic procedure combination, Japan

## Abstract

*Background and Objective:* The benefits of physical rehabilitation for very-low-birth-weight infants (VLBWI) have been reported in previous studies; however, the implementation rate of physical rehabilitation in this population remains to be clarified. This study aimed to examine the implementation rate of physical rehabilitation among VLBWI admitted to the neonatal intensive care unit (NICU) using real-world data. *Material and Methods:* This observational study obtained data from a nationwide administrative database associated with the diagnostic procedure combination (DPC) system in Japan (2014–2019). The participants were 30,464 infants admitted to the NICU between 2014 and 2019. The overall NICU physical rehabilitation rates and background factors of the participants were examined. *Results:* The overall physical rehabilitation rate in NICUs was 18%. Infants born at <28 weeks of age and extremely low birth weight infants (ELBWI) were more likely to receive physical rehabilitation interventions. The length of stay at the NICU and hospital, as well as the rate of discharge, were higher in patients who received physical rehabilitation than those in infants who did not. *Conclusions:* One-fifth of all patients admitted to the NICU received physical rehabilitation interventions. Extremely preterm infants and ELBWI were more likely to receive physical rehabilitation interventions. We need to consider ways to increase physical rehabilitation intervention rates in the NICU.

## 1. Introduction

The survival rates of extremely low birth weight infants (ELBWI) and very-low-birth-weight infants (VLBWI) have increased with recent advances in the field of medical technology [1]. The safest perinatal care system and lowest infant mortality rate based on survival period have been reported in Japan [2]. The neonatal mortality rate has been declining in recent years, reaching 0.79% according to a survey conducted in 2022. The total number of births has been declining annually in Japan; however, the proportion of VLBWI to total births has not shown any decrease [3].

Approximately 890,000 babies were born in Japan in 2019, according to the Japanese Vital Statistics. Among them, 9.4% were low-birth-weight infants. Among these very-low-birth-weight infants, 7.3% were VLBWI who were managed in the NICU after birth [2]. Various medical problems are observed in VLBWI. Moreover, VLBWI were found to gain less weight during hospitalization [4].

Neonatal hypoxic–ischemic encephalopathy, neonatal respiratory distress syndrome, patent ductus arteriosus, intraventricular hemorrhage, periventricular leukomalacia, and hydrocephalus can lead to hypoxic–ischemic encephalopathy in VLBWI and can cause other central nervous system disorders. Furthermore, the developmental prognosis of ELBWI is poorer than that of healthy term infants, with developmental defects and cerebral palsy often observed in addition to stunted growth [5,6].

Generally, the incidence of cerebral palsy is reported to be 1.5–3.4 per 1000 live births. However, the incidence of cerebral palsy in VLBWI has been reported as 1–4 per 1000 live births [7,8,9].

Additionally, long-term ventilation for the management of severe chronic lung disease is a significant risk factor for cerebral palsy [10]. Several studies have reported the effects of physical rehabilitation on the growth and development of VLBWI. Physical rehabilitation for VLBWI involves massage and stretching. Early commencement of interventions has been associated with better growth and development, including increased height, weight, and bone density [11,12,13,14,15]. In addition, some studies have reported that physical rehabilitation interventions are effective in reducing the duration of hospital stay by 5–10 days [16,17]. Physical therapy in the NICU is evidence-based, and positioning and handling of the infant by a specialized therapist, along with exercise therapy, are recommended [18,19]. Therefore, early physical rehabilitation intervention for VLBWI needs to be proactive because of its impact on developmental promotion and reduced length of hospital stay.

Physical rehabilitation for VLBWI has been performed worldwide, with many reports linking exercise in particular to growth and development. However, most interventions are performed by doctors, nurses, and mothers, and few studies involving rehabilitation professionals (physical therapists, occupational therapists, and speech-language pathologists) have been conducted. Thus, the status of the physical rehabilitation interventions performed by rehabilitation professionals in NICUs in Japan remains unknown. This study examined the rate of implementation of physical rehabilitation interventions by rehabilitation professionals in NICUs in Japan using real-world data.

## 2. Material and Methods

### 2.1. Study Design and Data Source

The diagnosis procedure combination (DPC) is a case-mix patient classification system launched by the Ministry of Health, Labour and Welfare of Japan in 2002. This method calculates medical fees by combining the comprehensive evaluation (including medication, injection, and hospitalization), which consists of a fixed number of points per day determined by the Ministry of Health, Labor and Welfare, and the piece rate evaluation (surgery, anesthesia, etc.) based on the presence or absence of medical services such as surgery based on the patient’s disease title and symptoms. DPC data belong to medical fee schedule databases, which include medical history data as well as some clinical data. 

This observational study was conducted using DPC data between 1 April 2014 and 31 March 2020. The DPC data were collected by a DPC research group and covered approximately 90% of tertiary emergency hospitals in Japan [20]. Details regarding the DPC data have been described in previous reports [21]. The DPC data include essential patient information such as sex, age, and admission and discharge routes; information pertaining to diagnoses, such as concurrent conditions upon admission and complications; and details of medical procedures performed during hospitalization, such as surgeries and physical rehabilitation. The DPC data can be used to analyze utilization, access, outcomes, and costs of health care services [22]. The DPC is one of the medical fee schedule databases, which includes detailed medical history data as well as some clinical data. Clinical research using this database has been actively conducted in recent years [23].

The study design was approved by the Ethics Committee of Medical Research, University of Occupational and Environmental Health, Japan, Japan (R4-045), which waived the requirement for in- formed consent.

### 2.2. Participants

**Figure 1:**
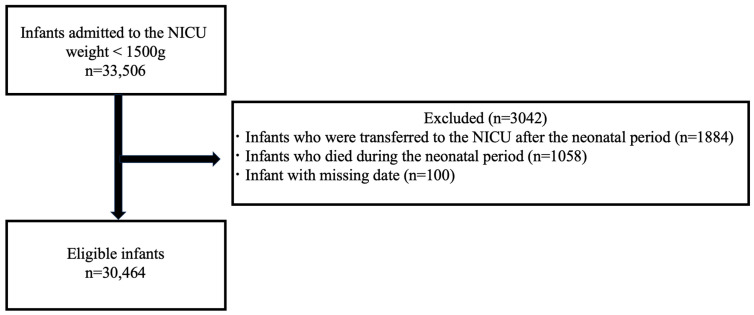
Flowchart of study inclusion.

A total of 33,506 infants weighing < 1500 g at birth who were admitted to the NICU were included in this study. The exclusion criteria were as follows: infants who were transferred to the NICU after the neonatal period (0–27 days after birth) (n = 1884), infants who died during the neonatal period (n = 1058), and infants with missing data (n = 100). Thus, 30,464 infants were included in the analysis after applying the exclusion criteria (Figure 1).

### 2.3. Physical Rehabilitation at NICU

Cases wherein a medical fee related to physical rehabilitation had been claimed at least once during the NICU stay were defined as having undergone physical rehabilitation (physical therapist [PT], occupational therapist [OT], speech therapist [ST]) in the NICU.

### 2.4. Other Variables

The sex, gestational age, birth weight, fiscal year, comorbidities at the time of admission, length of stay (LOS) at the NICU, LOS at the hospital, and discharge destination of the infants were examined. The infants were categorized as extremely pre-term (<28 w), very pre-term (28–31 w), moderately pre-term (32–33 w), late pre-term (34–36 w), or full-term/late term/post-matter/post-mature (>36 w) according to the gestational age. The infants were classified into the categories of <1000 g (extremely low birth weight [ELBW]) and 1000–1499 g (very low birth weight [VLBW]) according to the birth weight. The presence of comorbidities was determined on the basis of the International Classification of Disease, tenth edition (ICD-10) codes.

### 2.5. Statistical Analysis

LOS at the NICU and hospital are continuous variables and are presented as the median and interquartile range. Other variables are presented as categorical variables using numerical values and percentages.

Characteristics such as sex, gestational age, weight, and fiscal year of the infants in the group that received physical rehabilitation in the NICU were compared with those of the infants in the group without physical rehabilitation in the NICU using the Mann–Whitney U test and the chi-square test.

Diseases that occurred at a frequency of ≥1% were listed in descending order, and the number of occurrences and the prevalence rate were calculated to determine the concurrent conditions that patients had while undergoing physical rehabilitation in the NICU.

The time of initiating postnatal physical rehabilitation was examined for patients who underwent physical rehabilitation in the NICU, and the patients were categorized according to birth weight (ELBW or VLBW). Ten patients who started receiving physical rehabilitation >100 days after birth were excluded.

The LOS at the NICU and hospital, as well as the discharge destination of the 29,796 participants who were alive at the time of discharge, were determined. The patients who underwent physical rehabilitation in the NICU and those who did not were evaluated using the Mann–Whitney U test and chi-square test. All statistical analyses were performed using the Stata Statistical Software Release 18 (StataCorp LLC, College Station, TX, USA). Statistical significance was set at *p* < 0.05.

## 3. Results

Table 1 presents the participant characteristics. The number of patients with shorter gestation periods was higher in the group that received physical rehabilitation than that in the group that did not receive physical rehabilitation in the NICU (*p* < 0.001). Moreover, the proportion of ELBWI was significantly higher in the group that received physical rehabilitation (*p* < 0.001). Therefore, among infants who received physical rehabilitation interventions, extremely preterm and ELBWI infants may have received them more frequently.

Table 2 presents the comorbidities observed in the participants who underwent physical rehabilitation in the NICU. 

Neonatal respiratory distress syndrome was observed in half of the infants with VLBW. Patent ductus arteriosus, a respiratory circulatory disorder that requires physical rehabilitation, was observed in 20% of infants with VLBW.

Infants with cerebrovascular disorders, such as hypoxic–ischemic encephalopathy related to severe neonatal asphyxia, non-traumatic intraventricular hemorrhage, and periventricular leukoencephalopathy, also required physical rehabilitation. VLBWI with retinopathy of prematurity or neonatal seizures often presented with multiple comorbidities and often required physical rehabilitation interventions.

**Figure 2:**
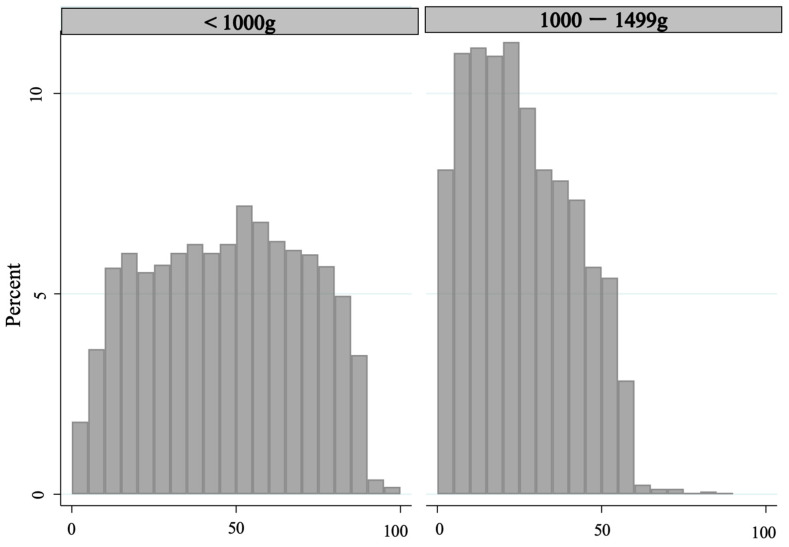
Days from birth to the start of physical rehabilitation in the NICU according to weight (n = 5630).

Figure 2 presents the timing of initiation of NICU physical rehabilitation based on birth weight. The median intervals to initiating physical rehabilitation in the NICU were 47 days (IQR: 26–66 days) and 23.0 days (IQR: 12.0–38.0 days) for ELBWI and VLBWI, respectively, indicating that physical rehabilitation was initiated significantly earlier for VLBWI than for ELBWI (*p* < 0.001).

Table 3 presents the results of the comparison of the LOS at the NICU and hospital, as well as the discharge destination, between the infants in the groups that did and did not receive physical rehabilitation in the NICU. The LOS at the NICU and hospital in the group that received NICU physical rehabilitation were significantly longer than those in the patients who did not receive physical rehabilitation in the NICU (*p* < 0.001). The rate of discharge to home was significantly higher in the group that received physical rehabilitation in the NICU than that in the group that did not receive physical rehabilitation in the NICU (*p* < 0.001).

## 4. Discussion

The present study, conducted using DPC data, is the first to reveal the proportion of physical rehabilitation professionals involved in the rehabilitation of infants admitted to the NICU, the duration of intervention, and outcomes for affected infants in Japan.

This 6-year study revealed that 30,464 infants were treated in the NICU within 28 days of delivery and that the rate of physical rehabilitation performed by rehabilitation professionals was 18% (n = 5640) of the total number of infants. Although the rehabilitation intervention rate of approximately 18% for VLBWI seems low, there is no clear curriculum for specialized rehabilitation for neonates, including training schools, and the skills can be acquired by participating in many specialized training programs. Thus, it is necessary to investigate how many professionals who can perform physical rehabilitation of newborns exist in hospitals with NICUs in the future.

Because neonatal hypoxic–ischemic encephalopathy tends to progress to severe neonatal paralysis, VLBWI are more likely to present with central nervous system damage than ELBWI. Therefore, rehabilitation professionals need to intervene early for VLBWI who may present with central nervous system disorders, such as cerebral palsy, and provide specialized rehabilitation for developmentally appropriate neurological disorders [11,12,13,24]. In particular, infants with other motor activities require intervention at an earlier stage because they are effective in promoting development.

Most VLBWI started receiving physical rehabilitation interventions within 50 days of admission to the NICU, whereas ELBWI started receiving rehabilitation interventions within 100 days, with a wide variation. There are no clear guidelines for initiating physical rehabilitation, and interventions are initiated when the physician determines that the general condition of the infant is stable and physical rehabilitation is necessary. Although some VLBWI requiring artificial respiration due to neonatal respiratory distress syndrome such as respiratory distress syndrome, interventions performed by rehabilitation professionals could be initiated within 50 days, owing to the short time required for their general condition to stabilize.

According to the results of patient outcomes, the LOS in the hospital and NICU for infants who received physical rehabilitation was predominantly longer than that of those who did not. The infants who were extremely preterm and ELBWI were more severely affected. It is thought that this is related to the time it took for their overall condition to stabilize.

This finding suggests that infants with severe comorbidities are more suitable candidates for physical rehabilitation. 

The rate of home discharge was significantly higher in infants who received physical rehabilitation than that in those who did not receive physical rehabilitation. No significant differences were observed in terms of the number of transfers to hospitals and nursing homes or others.

However, the longer hospital stay may have facilitated the preparation of medical equipment and the identification of a primary care physician, as well as the coordination of a complete post-discharge support system, including home care nurses and home physical rehabilitation. It is necessary to examine the reasons more clearly in the future as the survey suggested that the percentage of patients discharged to their homes was predominantly higher.

This study has several limitations. First, the type of physical rehabilitation provided was unclear. However, generally, exercise therapy, such as positioning, handling, stretching, and massage, are provided for the growth and development of VLBWI [10,11,12,13,14,18]. Additionally, physical rehabilitation for pulmonary rehabilitation problems is provided [25,26]. Thus, it is assumed that such physical rehabilitation was also provided to the VLBWI included in this study. Second, in this study, rehabilitation was defined as a case in which reimbursement was billed by a PT, OT, or ST. Therefore, even if rehabilitation is performed but reimbursement is not billed, the documentation suggests that rehabilitation was not performed. Therefore, the rehabilitation implementation rate in this study may have been underestimated. Third, it is unclear whether there are rehabilitation professionals (PT, OT, and ST) in hospitals with NICUs who can manage VLBWI. Lastly, the DPC data used in this study does not include data from all hospitals in Japan and may not completely reflect the entire country. However, the data used in this study include 90% of tertiary emergency hospitals and can generally be considered to reflect the situation in Japan as a whole. Globally, no previous reports have investigated the rate of rehabilitation implementation by rehabilitation professionals in NICUs using such a large dataset.

## 5. Conclusions

This observational study was conducted using 6 years of DPC data, and it demonstrated that physical rehabilitation was provided to 18% of all patients in the NICU. Infants born at <28 weeks and ELBWI weighing < 1000 g received further physical rehabilitation interventions. Greater variation in the timing of physical rehabilitation initiation was observed in ELBWI than that in VLBWI. The findings of the present study revealed that physical rehabilitation provided at NICU for VLBWI is not widespread in Japan. Therefore, in the future, it is necessary to educate physical rehabilitation professionals in the NICU about the implementation of physical rehabilitation for VLBWI.

## Figures and Tables

**Table 1:** Characteristics of the participants (n = 30,464).

	Rehabilitation at NICU		
	Without (n = 24,824)	With (n = 5640)	*p*-Value
Sex			
Male	12,459 (50.2%)	2872 (50.9%)	0.320
Female	12,365 (49.8%)	2768 (49.1%)	
Gestational age (w)			
Extremely pre-term (<28 w)	7726 (31.1%)	2143 (38.0%)	<0.001
Very pre-term (28–31 w)	11,224 (45.2%)	2601 (46.1%)	
Moderate pre-term (32–33 w)	3457 (13.9%)	529 (9.4%)	
Late pre-term (34–36 w)	2124 (8.6%)	310 (5.5%)	
Full-term/Late-term/Post-term/Post-mature (>36 w)	293 (1.2%)	57 (1.0%)	
Weight (g)			
<1000	9422 (38.0%)	2715 (48.1%)	<0.001
1000–1499	15,402 (62.0%)	2925 (51.9%)	
Fiscal year			
2014	4857 (19.6%)	723 (12.8%)	<0.001
2015	4677 (18.8%)	900 (16.0%)	
2016	4174 (16.8%)	925 (16.4%)	
2017	3862 (15.6%)	998 (17.7%)	
2018	3757 (15.1%)	1064 (18.9%)	
2019	3497 (14.1%)	1030 (18.3%)	

NICU, neonatal intensive care unit.

**Table 2:** Comorbidities in the participants who received physical rehabilitation interventions in the NICU (n = 5640).

	Freq.	%
Neonatal respiratory distress syndrome	3047	54.0
Severe neonatal asphyxia	1721	30.5
patent ductus arteriosus	1145	20.3
Mild and moderate neonatal asphyxia	1036	18.4
Hemorrhagic disorders of the fetus and neonate	920	16.3
Anemia in premature infants	649	11.5
Neonatal transient tachypnoea	647	11.5
Neonatal hypoglycemia	628	11.1
Neonatal asphyxia	581	10.3
Apnea	540	9.6
Respiratory disease	385	6.8
Neonatal hypocalcemia	371	6.6
Bacterial sepsis	368	6.5
Neonatal jaundice (details unknown)	302	5.4
Neonatal hypothermia	278	4.9
Multiple pregnancy	242	4.3
Residual fetal circulation	236	4.2
Constipation	236	4.2
Respiratory failure in newborns	226	4.0
Iron deficiency anemia	194	3.4
Retinopathy of prematurity	193	3.4
Disseminated intravascular coagulation	179	3.2
Neonatal heart failure	154	2.7
Hypogammaglobulinemia	146	2.6
Hypoxic–ischemic encephalopathy	139	2.5
Neonatal convulsion	129	2.3
Nontraumatic intraventricular hemorrhage	123	2.2
Chronic respiratory disease	102	1.8
Acute pharyngitis	99	1.8
Specific developmental disorder	97	1.7
Perinatal infection	97	1.7
Rh isoimmunization	92	1.6
Vitamin K deficiency	88	1.6
Kernicterus	87	1.5
Ventricular septal defect	79	1.4
Acidosis	75	1.3
Newborn jaundice	73	1.3
Neonatal conjunctivitis	69	1.2
Periventricular leukomalacia	66	1.2
Non-traumatic intracranial hemorrhage	65	1.2
Amniotic infection	62	1.1

NICU, neonatal intensive care unit; Freq., frequency.

**Table 3:** Outcomes of participants (n = 29,796).

	Rehabilitation in the NICU		
	Without (n = 24,258)	With (n = 5538)	*p*-Value
Length of stay, median (IQR)			
In the NICU	48.0 (29.0, 63.0)	60.0 (47.0, 85.0)	<0.001
In the hospital	72.0 (52.0, 104.0)	90.0 (65.0, 122.0)	<0.001
Discharge destination			
Home	7726 (31.1%)	2143 (38.0%)	<0.001
Other hospital	11,224 (45.2%)	2601 (46.1%)	
Nursing home/Others	3457 (13.9%)	529 (9.4%)	
IQR, interquartile range			
Patients discharged owing to death were excluded.			

## Data Availability

The datasets used and/or analyses conducted during the current study are not publicly available for ethical reasons. The datasets are available from the corresponding author upon reasonable request and with ethical approval.
